# Anti-Arthritic Effect of Chebulanin on Collagen-Induced Arthritis in Mice

**DOI:** 10.1371/journal.pone.0139052

**Published:** 2015-09-24

**Authors:** Yinglan Zhao, Fang Liu, Yao Liu, Dan Zhou, Qing Dai, Songqing Liu

**Affiliations:** 1 Department of Pharmacy, Southwest Hospital, Third Military Medical University, Chongqing, China; 2 The Traditional Chinese Medicine Hospital of Dianjiang County, Chongqing, China; Wayne State University, UNITED STATES

## Abstract

Rheumatoid arthritis is a chronic degenerative autoimmune disease characterized by persistent inflammation of synovial membranes, which leads to cartilage destruction and bone erosion. To date, there are no effective therapies to slow the progress of this degenerative condition. Here, we evaluate the anti-arthritic effect of chebulanin, an abundant anti-inflammatory agent isolated from *Terminalia chebula*, in collagen induced arthritis in DBA/1 mice by intragastric administration. Arthritic severity was scored by performing histopathological evaluation of the joints and measuring the expression of inflammatory cytokines and relative enzymes by immunohistochemical staining. In parallel, bone destruction and erosion were confirmed by micro-CT. Our data revealed that chebulanin significantly improved the severity of arthritis. Specifically, the histopathological characteristics of the tissues were improved and expression of TNF-α, IL-6, MMP-3 and COX-2 in the paws and joints of the treated mice decreased in a dose-dependent manner compared with control mice. Furthermore, micro-CT analysis revealed that chebulanin induced a dose-dependent reduction in cartilage destruction and bone erosion. Taken together, our findings suggest that chebulanin suppresses the expression of inflammatory mediators and prevents cartilage destruction and bone erosion in mice. Therefore, chebulanin is a strong therapeutic alternative for the treatment of RA.

## Introduction

Rheumatoid arthritis (RA) is a chronic autoimmune disease characterized by persistent inflammation of synovial membranes and progressive cartilage destruction and bone erosion [1]. Currently, 1% of the world population is affected by RA and the incidence of this degenerative disease keeps increasing [2, 3]. The pathophysiology of RA remains mostly unexplained, but inflammatory mediators such as TNF-α, IL-6 and COX-2 are known to play a pivotal role in the inflammation of synovial membranes and the bone destruction observed in RA [4]. Therefore, chemical agents that can mediate the down regulation of these inflammatory components may have potential for the treatment of RA.

There are currently three therapeutic options for RA: non-steroidal anti-inflammatory drugs (commonly known as NSAIDs), disease-modifying anti-rheumatic drugs, and glucocorticoids [5]. Despite all being effective in the treatment of RA, their application in the clinic is limited by the severity of the side effects following prolonged use [6]. Recently, some therapeutic alternatives for RA that target inflammatory cytokines, namely infliximab (anti-TNF-α antibody; [7–9]), etanercept (a recombinant TNF receptor fusion protein; [10]) and anakinra (a recombinant human IL-1 receptor antagonist; [11]) have shown promising results in clinical trials and have been approved for the treatment of RA. However, these new therapeutics are costly and have also shown some undesirable side effects which limits their use in the clinic. Therefore, finding new therapeutic options for RA remains a priority.

*Terminalia chebula retzius (TC)* is a traditional folk medicine that has been widely used in Asian countries for its anti-oxidant [12], anti-inflammatory [13, 14], anti-bacterial [15] and anti-arthritic [16, 17] properties. Three polyphenolic compounds have been isolated from the fruits of *TC*: chebulagic acid, chebulanin, and chebulinic acid [18, 19]. Previously, we have shown that chebulanin functions as an anti-inflammatory agent in a lipopolysaccharide-stimulated RAW 264.7 cell model (data not shown). The collagen-induced arthritis (CIA) animal model is generally considered an adequate representation of the pathogeneic processes of human RA and the DBA/1 mouse strain was considered as the “gold standard” [20, 21]. Here, we investigate the anti-inflammatory and anti-arthritic properties of chebulanin on a collagen-induced arthritis (CIA) model in DBA/1 mice.

## Materials and Methods

### Chemicals and reagents

Macroporous resins (DIAION HP20) were purchased from Mitsubishi chemical (Japan). The following antibodies were from Santa Cruz Biotechnology (USA): TNF-α antibody (sc-52746), IL-6 antibody (sc-1265), COX-2 antibody (sc-23984) and matrix metalloproteinase (MMP)-3 antibody (sc-6839). Soluble pure bovine type II collagen was purchased from Chondrex Inc. (USA). Complete Freund’s adjuvant and incomplete Freund’s adjuvant were purchased from Sigma-Aldrich (USA).

### Preparation and identification of chebulanin

Immature fruits of *TC* were obtained from the Guangxi Botanical Garden of Medicinal Plants (voucher specimen no. 6176) in May 2012 and were identified by Lanlan Fan (Guangxi Botanical Garden of Medicinal Plants, Guangxi Zhuang Autonomous Region of China, China). No endangered or protected species were involved in the study and no specific ethics protocols were required.

The dry fruits (400 g) of the plant were crushed and extracted three times with a 70% acetone solution (1:10, w/v) at room temperature (23 ±2°C) for 72 h. The supernatant was filtered, concentrated using a rotary evaporator under reduced pressure and lyophilized to yield a dry brown product (yield, 11.3%). The product was then dissolved in methanol and separated using a DIAION HP20 macroporous resin (Mitsubishi chemical) by different portion of methanol-water gradient. The fraction isolated by using a 40% methanol-water solution was concentrated and recrystallized, yield off-white amorphous powder. (yield, 3.5 g). Electrospray ionization-mass spectrometry (ESI-MS) and nuclear magnetic resonance analyses (^1^H-NMR and ^13^C-NMR) were used to confirm the purity and identity of this substance. For the mass spectrometry analysis, Agilent 6410 Triple-Quadrupole LC/MS coupled with an Agilent 1200 Series HPLC system was applied and operated in the negative ion modes. ^1^H and^13^C nuclear magnetic resonance (NMR) spectra were performed on Agilent 600 MHz NMR spectrometer.

### Animals

Six-week-old male DBA/1 mice (18 ± 2 g) were purchased from the Institute of Experimental Animals in the Third Military Medical University (China; rodent license no. SYXK (yu) 2012–0002). Animals were housed in specific pathogen-free conditions and provided with standard mouse chow and water *ad libitum*. All experimental procedures involving animals were approved by the Animal Research Ethics Committee of the Third Military Medical University and were performed in accordance with institute guidelines for humane and ethical care of animals. In order to reduce the suffering in mice, we invited an expert to help us induce the arthritis model (well-trained and experienced in the procedure). Furthermore, we chose a non-invasive intragastric route for drug administration, which lessened suffering. All efforts were made to reduce suffering of the animals and minimize the number of animals used in the study.

### Induction of CIA and treatment protocol

**Fig 1:**
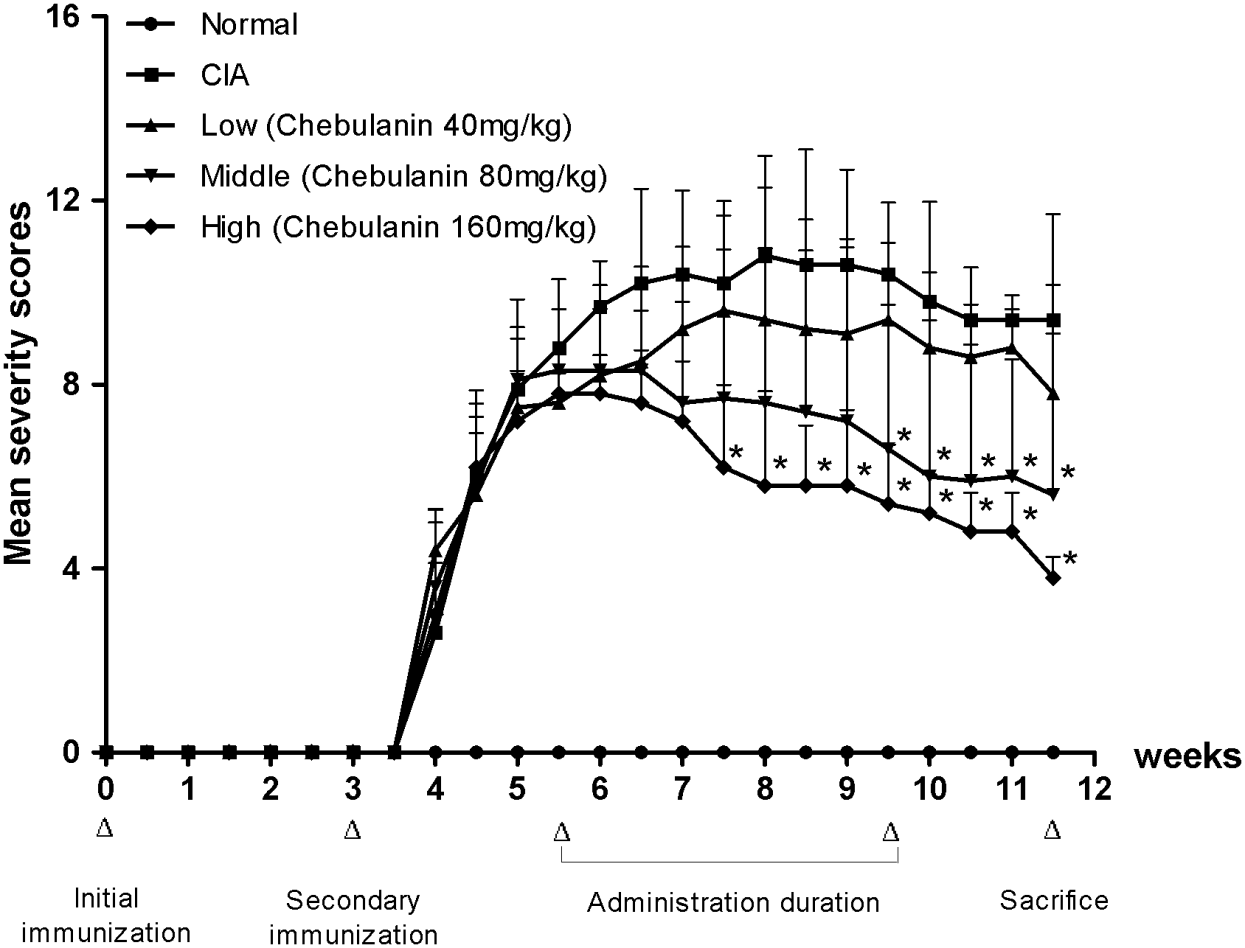
Treatment with chebulanin ameliorated the clinical severity of CIA. CIA was induced, and after the onset of arthritis animals were treated with vehicle (control) or chebulanin (at 40 mg/kg, 80 mg/kg or 160 mg/kg) daily for 28 days. Severity of arthritis was evaluated every two days. Error bars represent the standard deviation (n = 6); * *p* <0.05 vs. untreated CIA mice.

A total of 30 male DBA/1 mice were randomly divided into the following five groups (with six animals (n = 6) in each group): normal group = normal mice; CIA group = collagen-induced arthritis mice; low-dose group = chebulanin 40 mg/kg-treated CIA mice; mid-dose group = chebulanin 80 mg/kg-treated CIA mice; high-dose group = chebulanin 160 mg/kg-treated CIA mice. The dose and the route of chebulanin administration used in this study were chosen on the basis of preliminary experiments. CIA in mice was achieved as described by Brand et al [21]. Briefly, all mice outside the normal group were administered a dose of bovine type II collagen (100 μg) emulsified with complete Freund’s adjuvant intradermally at the base of the tail. Animals received a booster injection of bovine type II collagen (100 μg) emulsified with incomplete Freund’s adjuvant 21 days after the initial immunization. The chebulalin was dissolved in 1% sodium carboxyl methyl cellulose (1% CMC-Na water solution) and administered intragastrically once daily starting at two-and-a-half weeks (day 39) after the booster injection (after full development of CIA) for a duration of 28 days. The normal and the CIA groups received a 1% CMC solution administered in the same manner as a control. Mice were observed for two more weeks after administration and sacrificed by cervical dislocation at the end of experimentation (a total of 80 days). A time line diagram of flow chart can be seen in result part (Fig 1). Ankle joints in the paw were removed and fixed in 10% neutral buffered formalin.

### Assessment of arthritis severity

Mice were evaluated every two days using a scoring system to assess individual paws for arthritis severity (see Table 1) [21]. The arthritis severity scoring was performed by two independent observers and the identity of the samples was withheld to prevent bias.

**Table 1:** Severity scoring system.

Severity score	Degree of inflammation
0	No evidence of erythema and swelling
1	Erythema and mild swelling confined to the tarsals or ankle joint
2	Erythema and mild swelling extending from the ankle to the tarsals
3	Erythema and moderate swelling extending from the ankle to metatarsal joints
4	Erythema and severe swelling encompassing the ankle, foot and digits. Ankylosis of the limb might be present.

### Micro-CT evaluation

Micro-CT scans were performed at the Chongqing Key Laboratory for Oral Diseases and Biomedical Sciences (China). Briefly, the right hind paws and joints were scanned and then reconstructed into a 3D-structure using a VivaCT 40 Micro-CT instrument (Scanco Medical, Switzerland). Scan parameters were set as follows: energy = 70 kV, current = 114 μA, integration time = 250 ms and projections = 1200. Bone was segmented from soft tissue using the Scanco Image Processing Language software set to: threshold = 225, sigma = 0.8, and support = 1, as determined empirically from control specimens.

In order to quantitatively assess the level of bone changes, three relative parameters (bone volume (BV), bone surface area/ BV (BS/BV) and trabecular thickness (Tb.Th) were analyzed from the slice where the calcaneus first appeared to the distal end. BV and Tb.Th reflected the bone preservation in affected paws and joints, whereas BS/BV reflected the loss of bone surface due to erosion [22].

### Histopathological evaluation

Paws and joints were removed and fixed in 10% buffered neutral formalin (pH 7.4) for two days. Samples were decalcified in 10% EDTA for 30 days, during which the decalcification liquid was changed every three days. Decalcified tissues were embedded in paraffin blocks and stained with hematoxylin and eosin (H&E) to study the degree of synovitis, cellular infiltration and bone erosion using microscopy. Samples were analyzed by two independent pathologists and received a score between 0 and 3: 0 = no damage, 1 = mild synovitis with few focal infiltrates or slight cartilage erosion, 2 = moderate synovitis with obvious focal infiltrates and cartilage erosion, 3 = extensive infiltrates and bone or cartilage erosion [23]. The sample treatment protocol was withheld from the evaluators to prevent bias.

### Immunohistochemical examination

Tissue samples were counterstained with H&E staining and incubated with specific antibodies for murine TNF-α, IL-6, COX-2 and MMP-3, followed by incubation with the appropriate secondary antibodies. Staining for the specific expression marker was scored by two pathologists and received a score between 0 and 3: 0 = no expression, 1 = mild expression, 2 = moderate expression, 3 = strong expression. Again, the sample identity was withheld to prevent bias.

### Statistical analysis

Results are expressed as mean ± standard deviation (SD) and significance was assessed using the version 17.0 Statistical Package of Social Sciences Program (SPSS) for Windows (USA). GraphPad Prism 5.0 software was used to construct the graphs. Comparative analysis of arthritis severity score, semi-quantitative histopathological analysis and immunohistochemical examination between two groups were evaluated by the Mann-Whitney U-test. Comparative analysis of parameters in micro-CT was performed using a one-way analysis of variance (ANOVA) and significance was set at a *p*-value <0.05. The Tukey multiple comparison test was used to establish significance between different testing groups.

## Results

### Isolation of chebulanin

**Fig 2:**
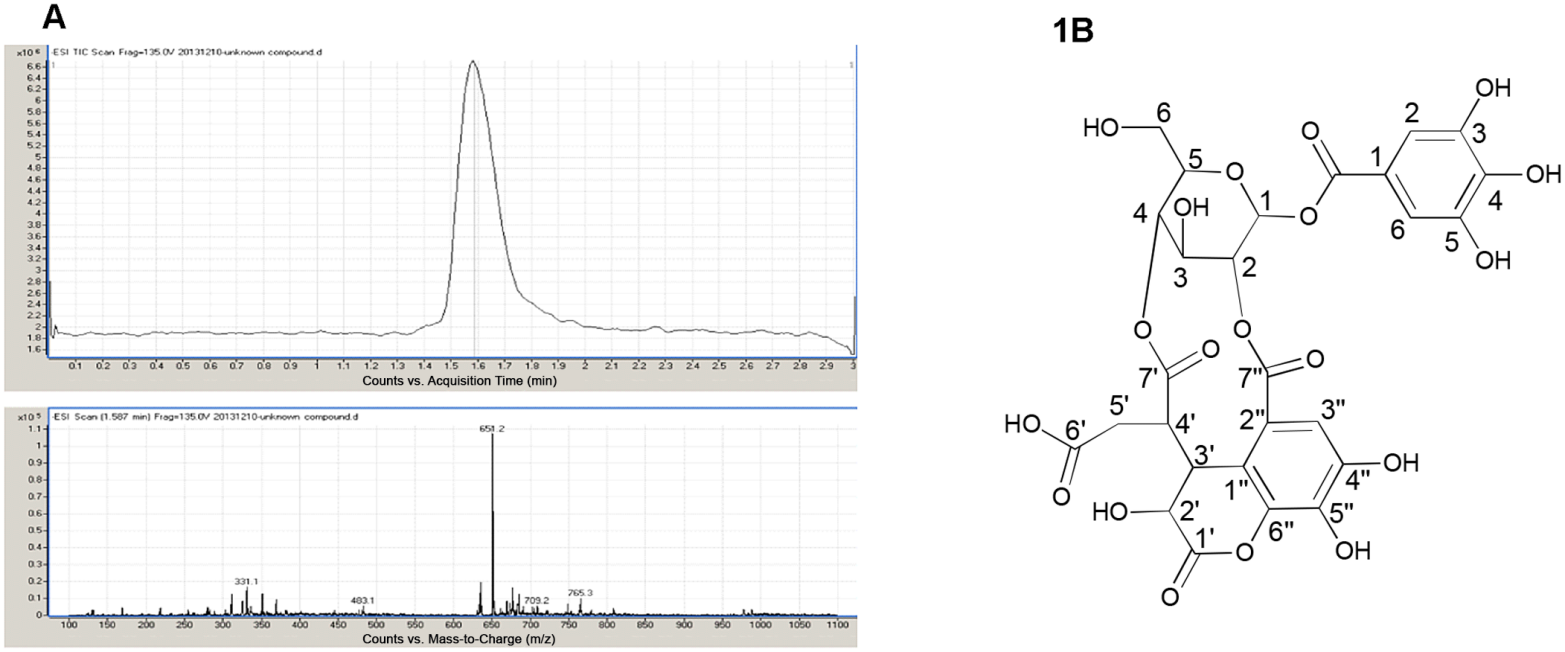
Identification and isolation of chebulanin. A: ESI-MS chromatogram of chebulanin isolated from immature fruit of *Terminalia Chebula*. B: Chemical structure of chebulanin.

An off-white amorphous powder that showed a dark blue color with ferric chloride reagent was purified from the immature fruits of *TC*. The sample was identified as chebulanin using ESI-MS, ^1^H-NMR and ^13^C-NMR techniques. A molecular formula of C_27_H_24_O_19_ was calculated from the mass spectra [M^-^ m/z 651] obtained using ESI-MS. The ^1^H-NMR and ^13^C-NMR data confirmed the identity of chebulanin (Fig 2) [17, 24]. The identity of the ^1^H-NMR (MeOH-d4, 600MHz) signals were as follows: δ7.12 (2H,s, galloyl H-2, 6), 6.36 (1H, brs, glucose H-1), 5.21 (1H,brs, glucose H-2), 4.81 (1H,brs, glucose H-3), 4.86 (1H,d, J = 7.6Hz, glucose H-4), 4.32 (1H,t, J = 6.5Hz, glucose H-5), 4.07 (1H,dd, J = 11.2,6.5Hz, glucose H-6a), 4.02 (1H,dd, J = 11.2,6.5Hz, glucose H-6b), 4.79 (1H,d, J = 7.2Hz,chebuloyl H-2’), 5.10 (1H,d, J = 7.2Hz,chebuloyl H-3’), 3.81 (1H,m, chebuloyl H-4’), 2.15 (2H,m, chebuloyl H-5’), 7.46 (1H,s, chebuloyl H-3”). The ^13^C-NMR (Acetone-d6, 600MHz) signals were assigned as follows: δ118.93 (galloyl C-1), 108.68 (galloyl C-2,6), 144.66 (galloyl C-3,5),138.10 (galloyl C-4), 163.88 (galloyl–COO-), 91.01 (glucose C-1), 72.23 (glucose C-2), 59.92 (glucose C-3), 70.10 (glucose C-4), 77.61 (glucose C-5), 61.41 (glucose C-6), 168.16 (chebuloyl C-1’), 65.12 (chebuloyl C-2’), 39.46 (chebuloyl C-3’), 37.96 (chebuloyl C-4’), 28.77 (chebuloyl C-5’), 171.47 (chebuloyl C-6’), 172.51 (chebuloyl C-7’), 115.38 (chebuloyl C-1”), 117.72 (chebuloyl C-2”), 114.46 (chebuloyl C-3”), 145.04 (chebuloyl C-4”), 137.97 (chebuloyl C-5”), 139.48 (chebuloyl C-6”), 164.48 (chebuloyl C-7”).

### Chebulanin ameliorated the severity of arthritis in CIA mice

The severity of arthritis was evaluated subjectively using a scoring system. CIA developed gradually in DBA/1 mice after booster injection, leading to a 100% incidence of CIA at day 38. The severity of arthritis in CIA group increased in a time-dependent mode duration treatment stage, showing a significantly higher severity score than the normal group. Importantly, treatment with chebulanin significantly decreased the severity of arthritis in a dose-dependent manner, specifically at doses of 80 and 160 mg/kg (*p* = 0.015 and 0.001, respectively) (Fig 1). Treatment with chebulanin at a dose of 40 mg/kg also ameliorated the severity of arthritis but the improvement observed was not statistically significant (*p* = 0.207).

Administration of chebulanin did not affect body weight or have deleterious side effects in the mice during treatment (data not shown), suggesting that chebulanin is safe for this disease model at the concentrations used in this study.

### Chebulanin treatment prevents bone destruction in CIA mice

**Fig 3:**
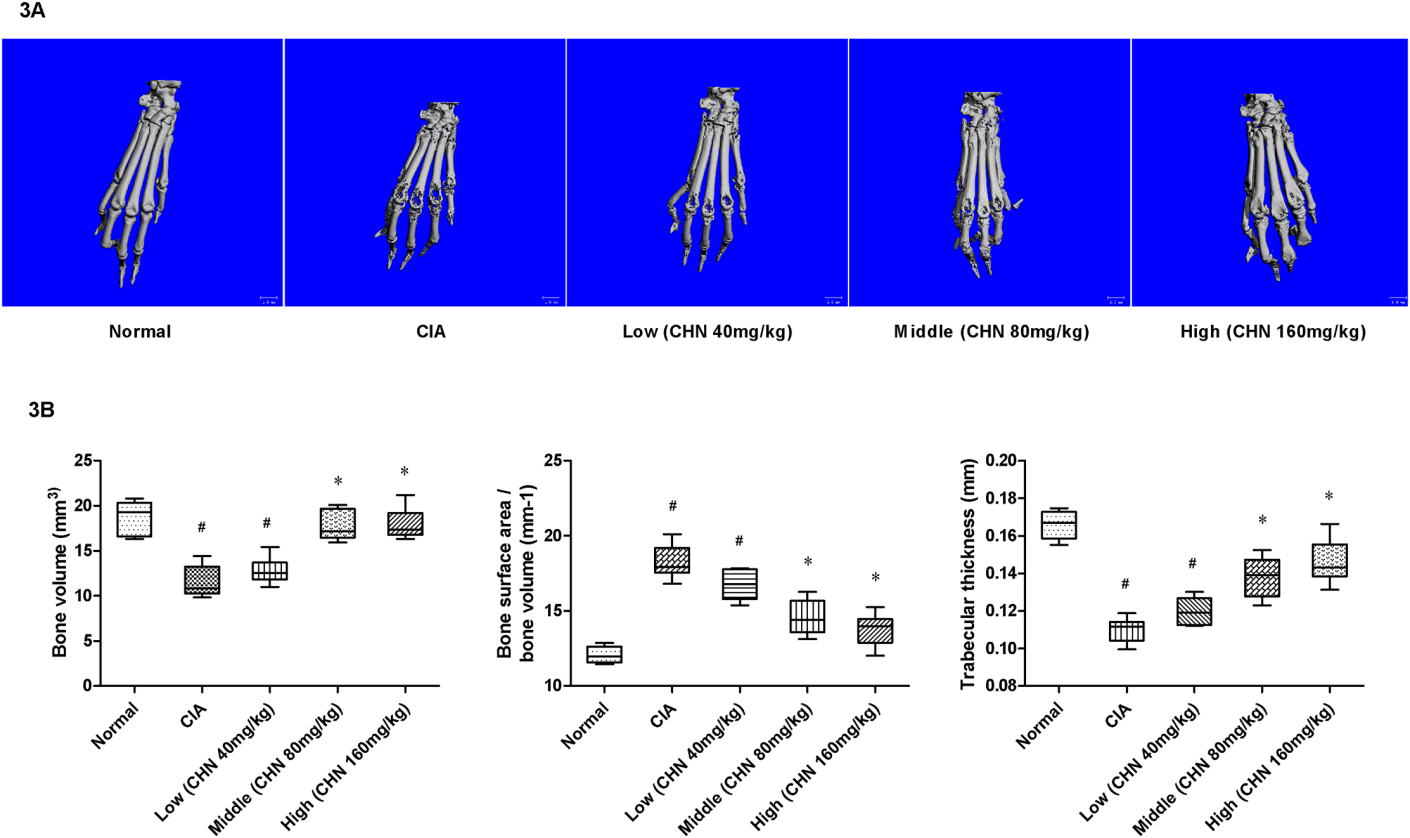
3D reconstruction of micro CT images of the hind paws and relative parameters of bone integrity. A: Representative 3D-micro computed tomography images of the hind paws showing joints from normal mice having smooth bone surface with intact joint architecture, and joints from CIA mice having significant bone erosion. B: Parameters of bone integrity. BV: bone volume; BS/BV: bone surface area/bone volume; Tb.Th: trabecular thickness. Data are expressed as mean ±standard deviation(n = 6); *p <0.05 vs. untreated CIA mice, ^#^ *p* <0.05 vs. normal mice.

The severity of joint destruction was assessed using 3D micro-CT (Fig 3). The paws of normal mice had a smooth bone surface, while those from untreated CIA mice had severe bone erosions. Importantly, chebulanin-treated CIA mice showed markedly less joint destruction, especially at the mid- and high-treatment doses of 80 mg/kg and 160 mg/kg.

Quantitative analysis of the BV, BS/BV and Tb.Th parameters showed that BV and TB Th were present at a higher volume in the chebulanin-treated mice than in the controls, and that this effect was dose-dependent. Similarly, the BS/BV in chebulanin-treated CIA mice was significantly lower than in untreated mice, indicating that chebulanin has a protective effect against bone damage.

### Chebulanin treatment improved the tissue architecture of paws and joints in CIA mice

**Fig 4:**
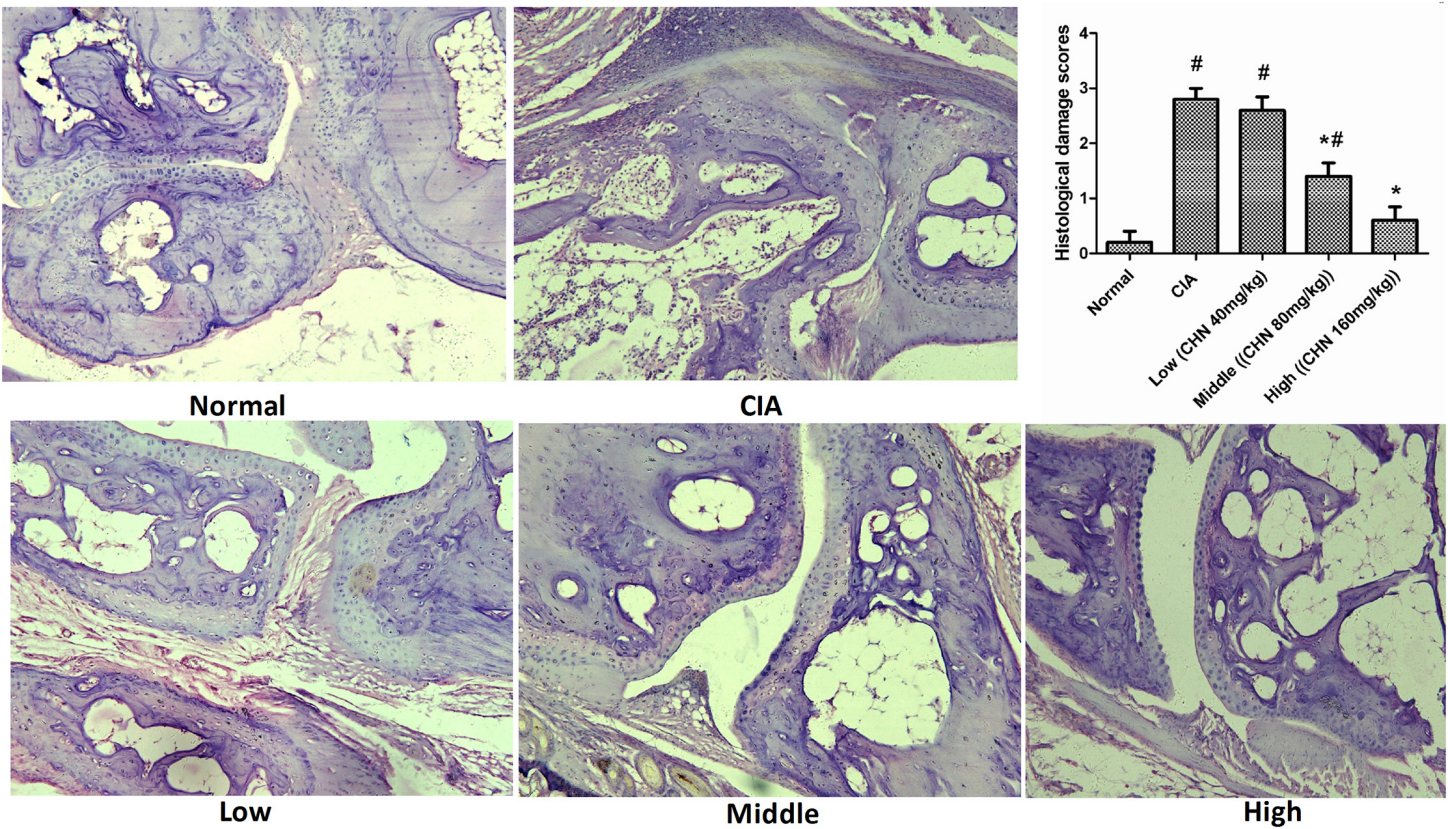
Hind paws of chebulanin-treated mice show improved bone architecture. H&E stained histopathological images of the hind paws from chebulanin-treated and control mice (40x). Semi-quantitative analysis of histopathological findings of the inflamed joints. Data are expressed as mean ± standard deviation(n = 6). *p <0.05 vs. untreated CIA mice, ^#^ *p* <0.05 vs. normal mice.

Histopathological assessment of paws and joints from treated and control mice revealed remarkable signs of cellular infiltration, synovial hyperplasia, pannus formation, partial cartilage and bone destruction in the untreated CIA mice. However, chebulanin-treated mice had significantly decreased levels of cellular infiltration, hyperplasia and bone destruction and this improved histopathology was dose-dependent. The semi-quantitative analysis of histopathological findings including infiltration and erosion scores is shown in Fig 4.

### Chebulanin decreased the expression of inflammatory cytokines and relative enzymes in inflamed joints of CIA mice

**Fig 5:**
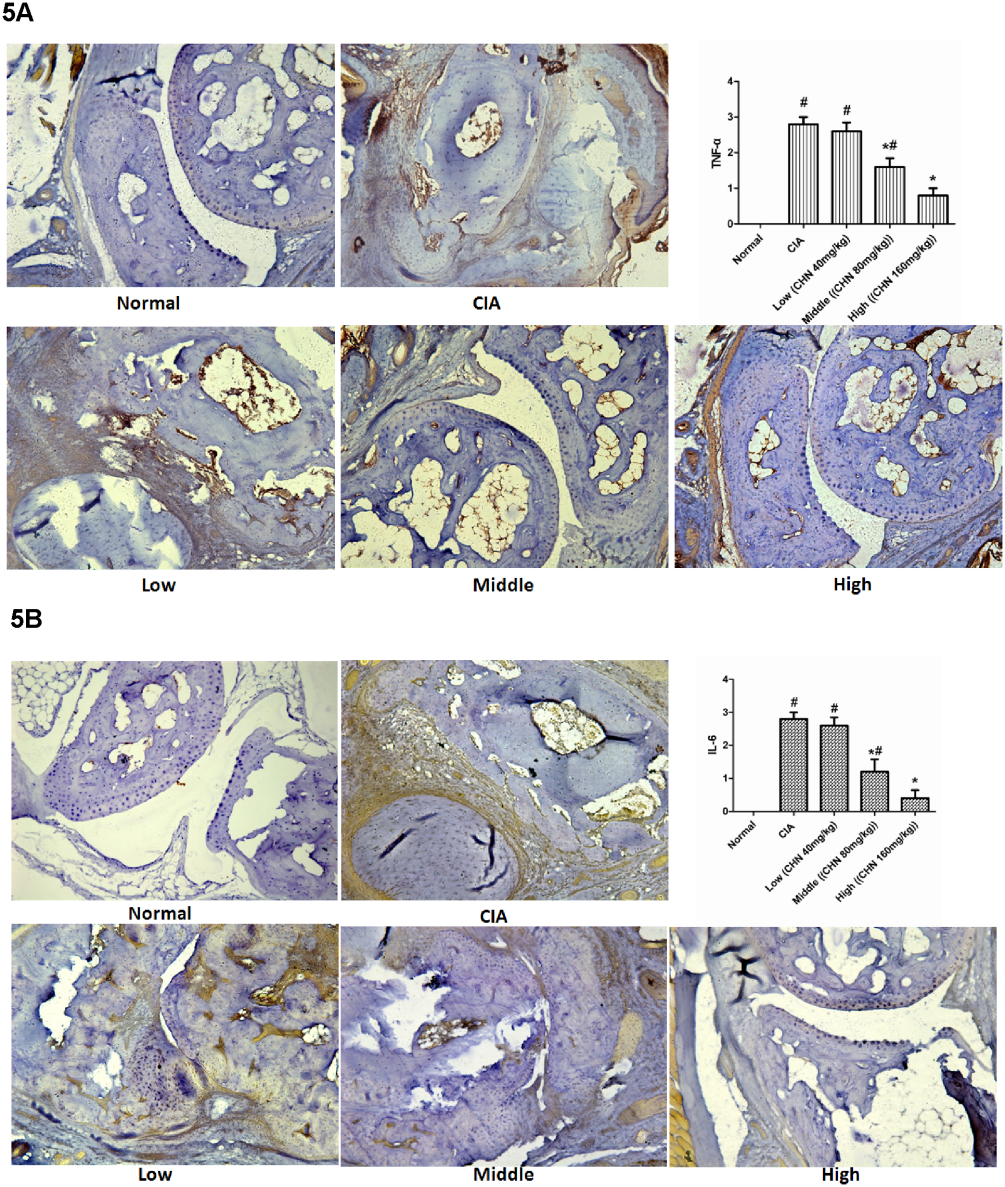
Chebulanin treatment decreased the expression of inflammatory cytokines in CIA mice. A: Immunohistochemistry analysis of TNF-α expression in the hind paws of mice and semi-quantitative analysis of staining score. B: Immunohistochemistry analysis of IL-6 expression in the hind paws and semi-quantitative analysis of staining score. All photomicrographs were obtained at 40x magnification and data are expressed as mean ± standard deviation (n = 6). *p <0.05 vs. untreated CIA mice, ^#^ *p* <0.05 vs. normal mice.

Immunohistochemical analysis of paws and joints in untreated CIA mice revealed remarkable expression of the inflammatory cytokines TNF-α (Fig 5A) and IL-6 (Fig 5B). In contrast, expression levels for these cytokines were significantly reduced following chebulanin treatment and undetectable in control mice.

**Fig 6:**
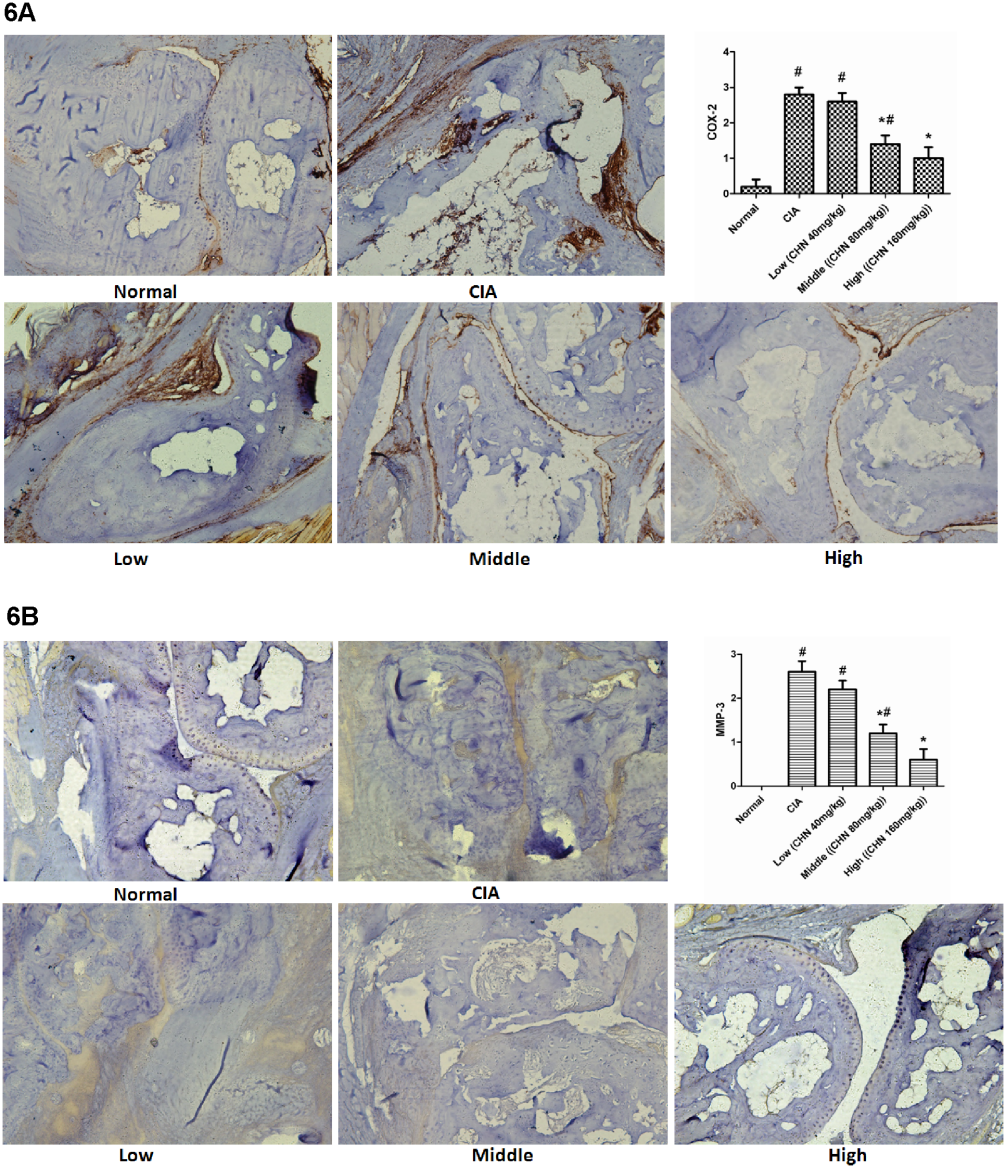
Chebulanin treatment decreases the expression of the inflammation- related enzymes COX-2 and MMP-3 in CIA mice. Immunohistochemical staining of (A) COX-2 and (B) MMP-3 expression in the hind paws of treated and control mice and semi-quantitative analysis of the staining score. Photomicrographs were obtained at 40x magnification and data are expressed as mean ± standard deviation (n = 6). *p <0.05 vs. untreated CIA mice, ^#^ *p* <0.05 vs. normal mice.

Similar results were obtained for COX-2 (Fig 6A) and MMP-3 (Fig 6B) expression. Semi-quantitative pathological analysis confirmed that the expression levels for these two markers were markedly reduced in chebulanin-treated mice compared to untreated CIA mice and that this effect was dose-dependent.

## Discussion

RA is a chronic autoimmune disease with a complex pathophysiology for which there is a limited array of therapeutic alternatives [1]. The CIA animal model is characterized by arthrocele, synovitis, cartilage destruction and bone erosion [25], and is generally considered an adequate representation of the pathogeneic processes of human RA [20, 26]. It has been demonstrated that several cytokines appear to direct cell to cell communication during the progression of CIA such as IL-1 [27], TNF-α [28]and IL-6 [27]. Here, we used the CIA model to test the anti-inflammatory and anti-arthritic properties of chebulanin for treatment of arthritis-related bone destruction. We found that chebulanin treatment reduced the clinical severity score of CIA in a dose-dependent manner. Furthermore, chebulanin treatment decreased the expression of the autoimmune disease-related cytokines (TNF-α and IL-6) and enzymes (COX-2 and MMP-3), indicating that the therapeutic mechanism of chebulanin may involve inhibition of inflammatory signaling in the joints of CIA mice.

Several pro-inflammatory cytokines have been characterized as important factors of RA pathogenesis [29]. Specifically, TNF-α has been shown to mediate an array of immune effector functions that induce the inflammatory response and pannus formation, supporting the cartilage destruction and bone erosion of RA [30]. In addition, TNF-α mediated activation of the transcription factor nuclear factor (NF-κB) establishes a positive feedback loop of this destructive cycle [31]. The pro-inflammatory cytokine IL-6 has also been shown to induce pannus proliferation, thereby promoting synovitis by recruiting inflammatory cells which leads to synovial hyperplasia. IL-6 can also promote the expression of various MMPs in synovial cells and chondrocytes which leads to cartilage destruction [32].

Current immunotherapeutic strategies in clinical use for RA treatment rely upon antibodies to TNF-α and the IL-6 receptor, as well as other bispecific antibodies or antibody multimers [9, 33–36]. Unfortunately, the immunotherapy treatment option is expensive and inconsistent (i.e. not every patient responds or to the same extent), and prolonged use is associated with severe adverse reactions. Safer, more affordable therapeutics for RA may be found among natural therapeutic agents, such as plant-based compounds. Herein, we describe the anti-arthritic and anti-inflammatory properties of chebulanin in a well-established mouse model that reflects many of the physical and molecular pathogenic properties of human RA.

RA progression in humans is accompanied by alterations in bone architecture, thus our analysis of the therapeutic potential of chebulanin included investigation of protection against CIA-related changes in bone integrity. These analyses were carried out using 3D micro-CT imaging, which allows for reconstruction of bone surfaces and quantitative measurement of disease- and treatment-related structural alterations in bone. The chebulanin treatment led to higher total bone volume, a greater extent of bone preservation, less loss of bone surface due to erosion less extensive osteopenia in the affected joints of the treated CIA mice, compared to their untreated CIA counterparts.

Analysis of the underlying molecular mechanisms of chebulanin-related protection against loss of bone integrity found that the chebulanin treatment led to suppression of expression of the proinflammatory cytokines TNF-α and IL-6 in the arthritic joints of the CIA mice, in dose-dependent manners and without serious side effects. The chebulanin treatment was also found to lead to significant inhibition of the expression of COX-2, an enzyme that converts arachidonic acid to PGE_2_, itself a mediator of inflammation, joint pain, and cartilage damage [37]. Finally, the CIA-related elevation in expression of MMP-3 in paws and joint tissues was found to be significantly decreased following the chebulanin treatment. MMPs are a group of zinc-dependent endopeptidases that are crucial for normal turnover of extracellular matrix, but which can also play a pathogenic role in RA [38], and elevated MMP-3 expression has been shown to be associated with the process of collagen destruction in cartilage [39, 40].

**Fig 7:**
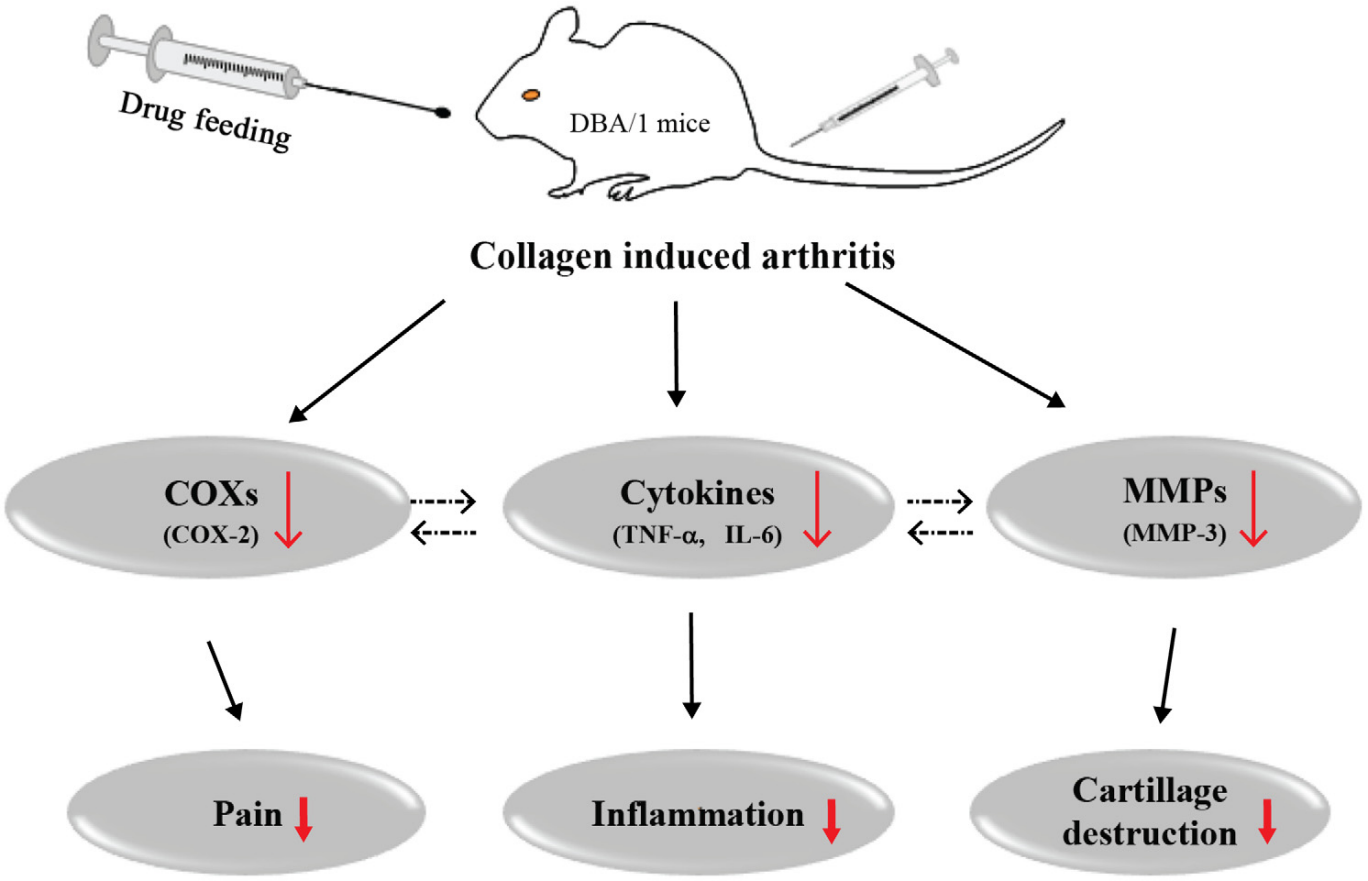
Chebulanin suppresses CIA in mice by different mechanisms. Chebulanin treatment ameliorates collagen-induced arthritis by inhibiting the expression of pro-inflammatory cytokines (TNF-α, IL-6), cyclooxygenase (COX-2) and matrix metalloproteinases (MMP-3).

The fruits of *TC* have been widely used in many Asian countries as a traditional folk medicine for treating cough or diarrhea. The *TC* bioactive constituents include the polyphenols chebulagic acid, chebulinic acid, chebulanin and gallic acid [17], which have anti-oxidant [12], anti-inflammatory [13, 14], anti-bacterial [15] and anti-arthritic properties [16]. Previously, we demonstrated the *in vitro* anti-oxidative and anti-inflammatory properties of the chebulanin polyphenol. In this study, we show that oral administration of chebulanin to CIA mice efficiently alleviates the severity of arthritic bone features. The mean arthritis score in our chebulanin-treated CIA mice was found to be significantly lower than that of the untreated CIA mice, especially at doses of 80 mg/kg and 160 mg/kg. In addition, chebulanin significantly improved the CIA-related histopathological changes through a mechanism involving decreasing the expression of inflammatory cytokines (TNF-α, IL-6) and inflammation-associated enzymes (COX-2, MMP-3) in joint tissues. Importantly, no important adverse effects were observed in the mice following the chebulanin treatment. In summary, we show for the first time that chebulanin has therapeutic potential for the treatment of RA based upon our observations that it can improve CIA by suppressing inflammatory mediator release and preventing cartilage and bone destruction (Fig 7).
